# Termination of pregnancy due to Thalassemia major, Hemophilia, and Down's Syndrome: the views of Iranian physicians

**DOI:** 10.1186/1472-6939-9-19

**Published:** 2008-12-23

**Authors:** Mehran Karimi, Mohammadmehdi Bonyadi, Mohhamad reza Galehdari, Soheila Zareifar

**Affiliations:** 1 Thrombosis and Hemostasis Unit, Hematology Research Center, Dept. of Pediatrics, School of Medicine, Shiraz University of Medical Sciences, Shiraz, Iran

## Abstract

**Background:**

Genetic disorders due to **kindred **marriages are common medical conditions in Iran; however, the legal aspects of abortion remain controversial. This study was undertaken to determine physicians' opinions regarding the termination of pregnancy for three genetic diseases: thalassemia major, hemophilia, and Down's syndrome.

**Methods:**

A questionnaire was administered to selected physicians by stratified random sampling to determine the following: age, gender, knowledge about prenatal diagnosis of diseases in high risk pregnancies, agreement with abortion, recommended gestational age for abortion, and, if opposed to abortion, the reason.

**Results:**

Of 323 physicians, who participated in the study, 91.3(295), 40.6(131), and 78.6%(254) were in agreement and 8.7(28), 59.4(192), and 21.4%(69) were opposed to abortion for thalassemia major, hemophilia, and Down's syndrome, respectively. Among 289 physicians opposed to abortion in respect of each of all three conditions, the following reasons were cited: religion, 18; emotional, 10; quality of care, 23; hope to find a new treatment option in the future, 103; miscellaneous reasons, 6; and a combination of these reasons, 129. Among 680 physicians in agreement with abortion in relation to all of the diseases, 4.6%(31) were agreed with abortion in less than 12 weeks gestation, 79.2%(538) in less than 16 weeks gestation, 5.6%(38) in less than 20 weeks gestation, 2.2%(15) in less than 24 weeks gestation, and 8.4%(58) were agreed with beyond the 24 weeks of gestational age.

**Conclusion:**

The majority of physicians were in agreement with abortion for thalassemia major and Down's syndrome because of the overall prognosis, but opposed to abortion for hemophilia.

## Background

Genetic disorders are common medical conditions, one of which is the thalassemia syndrome, a heterogeneous group of inherited anemias characterized by defects in the synthesis of one or more of the globin chain subunits of the hemoglobin tetramer [1]. Thalassemia syndrome is seen in all ethnic groups and geographic areas, but is most common in the Mediterranean basin[1]. The so-called thalassemia belt extends along the shores of the Mediterranean and throughout the Arabian Peninsula, Turkey, Iran, India, and southeastern Asia (Thailand, Cambodia, and southern China) [1]. Skeletal changes, hepatomegaly, cardiac dilation, small airway obstruction, enlargement of the kidneys, pigmentory gallstones, and growth retardation are reported to result from the disease [1]. Hemophilia, a sex-linked hereditary coagulopathy, occurs due to the deficiency of factors VIII and IX (hemophilia A and B, respectively). The hallmark sign of hemophilia is painful bleeding into the joints, leading to chronic inflammation [2].

The most common genetic disease in humans is Down's syndrome, with an incidence of 1 in 650–700 at birth. It has a strong association with an increase in maternal age [3]. Trisomy 21, an additional chromosome of maternal origin in over 90% of cases, leads to facial, limb, cardiac, and digestive tract abnormalities [3]. The field of prenatal diagnosis has emerged and been refined in the past 3 decades, with techniques that differ in terms of indications, invasiveness, complications, and risks, such as amniocentesis, [3,4] cordocentesis, [3,4] PGD(Preimplanation Genetic Diagnosis) [5] and PCR.(**Polymerase chain reaction) **[6,7].

Other studies by Julian et al. reported 78% acceptance rate for abortion in Down's syndrome and 21% agreement for abortion in hemophilia [8].

Up to 10% of families with a child with a genetic disorder are unable or unprepared to cope with such a child [3-5]. The presence of genetic abnormalities in the fetus in the majority of developed countries is an acceptable legal indication for termination of pregnancy [5]. Limitations of our study was government disagreement with abortion for all three diseases, thus may be interfere to make decision of physician regarding abortion in these cases. But the strength point of study was well familiarity of these diseases for our physician because they are not rare in Iran.

This study was undertaken to determine the opinions of Iranian physicians for termination of pregnancy in mothers with a prenatal diagnosis of thalassemia major, hemophilia, and Down's syndrome.

## Methods

This cross-sectional and descriptive study was conducted in southern Iran during 3 months (October to December) in 2005. A questionnaire was provided (see Additional file 1) to determine the opinions of physicians at Shiraz University of Medical Sciences in Shiraz, Iran regarding abortion for three genetic diseases (thalassemia major, hemophilia, and Down's syndrome). Age, gender, the feasibility of performing prenatal diagnosis (PND) for genetic disorders, agreement with abortion, the reasons for positive response (outcome of the disease, religious permission of abortion, poor feasibility of treatment, severity of the disease and Miscellaneous), gestational age at the time the abortion was recommended, and reasons (if any) upon which their disagreement with abortion (emotional, religious, quality of care, and hope for treatment options in the future) was based, were all considered. This research was approved by the Medical Ethics Committee of Shiraz University of Medical Sciences. Written informed consents were obtained from all physicians.

Of 770 physicians in 10 departments, 384 were selected in this study anonymously by stratified random sampling. (P = 0.5, d = 0.05, confident interval=%95) Out of 384, 323 physicians (response rate: 84%) participated in this study. They were studying in Shiraz University of Medical Sciences and were belonged to the different parts of Iran. On the basis of the number of physicians in each ward, sample size allocated to each ward by stratified random sampling. They were in 3 degrees: Intern, Resident and attending. The departments to which the physicians were attached included Internal Medicine, Surgery, Pediatrics, Obstetrics and Gynecology, Community Medicine, Psychiatry, Pathology, ENT, Ophthalmology, and Radiology. Completion of the questionnaire required 20–30 minutes and it covered the opinion of the responding physician regarding the view of abortion for thalassemia major, hemophilia, and Down's syndrome.

All the data were analyzed using SPSS software version 11.5 and chi square Statistical analysis in our qualitative data was done. The P value less than 0.05 was considered significant.

## Results

Among the respondents, 201 (62.2%) were males and 122 (37.8%) were females. Among them, 164 (50.8%) were interns, 106 (32.8%) were residents, and 53 (16.4%) were attending physicians.

Two hundred sixty-one (80.8%), 125 (38.7%), and 171 (52.9%) of the physicians were familiar with the prenatal diagnosis of thalassemia, hemophilia, and Down's syndrome, respectively. Two 295 (91.3%), 131 (40.6%), and 254 (78.6%) physicians were in favor of abortion for thalassemia, hemophilia, and Down's syndrome, respectively, while 28 (8.7%), 192 (59.4%), and 69 (21.4%) physicians, respectively, were opposed. The most common reason for agreement in abortion regarding thalassemia major and Down syndrome (72% and 80% respectively) were poor outcome and prognosis of the disease.

Among 289 physicians opposed to abortion in respect of each of all three conditions all of the three conditions, the following reasons were cited: religion, 18; emotional, 10; quality of care, 23; hope to find a new treatment option in the future, 103; miscellaneous reasons, 6; and a combination of these reasons, 129. Among 680 physicians in agreement with abortion in relation to all of the diseases, 4.6%(31) were agreed with abortion in less than 12 weeks gestation, 79.2%(538) in less than 16 weeks gestation,5.6%(38) in less than 20 weeks gestation, 2.2%(15) in less than 24 weeks gestation, and 8.4%(58) were agreed with beyond the 24 weeks of gestational age. The results of physicians in agreement with abortion by gestation for each of all three conditions (less than 12 weeks gestation, less than 16 weeks gestation, less than 20 weeks gestation, less than 24 weeks gestation, and beyond the 24 weeks of gestational age) were evaluated & were not significant(P > 0.05).

## Discussion

Our results showed that 91.3% of physicians agreed with abortion in the case of a prenatal diagnosis of fetal thalassemia, which the most common reasons were poor outcome and the severity of the disease, as well as the fact that thalassemia is a common genetic disorders in Iran and because of religious legal permission for abortion (Fatwa) by Ayattolahs before the 16^th ^week of gestation. Acceptability of abortion for Down's syndrome among the physicians was 78.6%, which is in agreement with the same findings of thalassemia major. Agreement with abortion for hemophilia was 40.6%, which is less than thalassemia major and down syndrome The opposition to abortion for hemophilia was nearly similar to the results of other studies [8,9] showing that the better outcome and prognosis of the disease do not justify the termination of pregnancy.

In Pakistan, majority of various social groups including lawyers, parents of thalassemic children and members of parliament were in favor of genetical diseases and abortion whereas only 24% of doctors favored making genetic screening [10].

The study showed a twice greater previously reported rate of willingness by Julian et al. [8] with abortion for hemophilia. Such a greater rate might be due to treatment and economic problems in our country. In other studies, the results showed that most physicians were in favor of abortion in Down's syndrome which is similar to our result [11].

In the study by Julian et al. [8] 994 physicians participated in the study and 687 (69%) completed the questionnaire. They were in agreement with abortion in the first trimester which is nearly similar to our study (83.8% < 16 weeks of gestation). Acquiring verbal and written consent for PND screening to obtain genetic information can disclose genetic information and genetic anomalies and help parents to overcome controversial issues of therapeutic abortion [12]. Physicians have a legal responsibility to discuss the implications of PND [13] and provide clear legal guidelines in their practice. Elkins et al. [14] mentioned counseling and obtaining informed consent from the patients for any procedure involving PND. In a study by Karimi et al. [9] comparing the attitudes towards PND and termination of pregnancy for hemophilia in Iran and Italy, 84.7% of the Iranians and 35.4% of the Italians were not familiar with the possibilities afforded by PND for hemophilia. Termination of pregnancy appeared to be accepted by 58.2% of the Iranians and 16.7% of the Italian participants. The results are different from our study in relation to hemophilia, however, we evaluated the physicians' views in relation to the acceptability of abortion and in the study of Karimi et al. [9], the mothers' views were evaluated. In Iran, we do not have permission to do abortions in cases of hemophilia, but our physicians hope to obtain permission to do so in the future.

## Conclusion

The majority of our physicians agreed with abortion in cases of thalassemia major and Down's syndrome. It Could be due to widespread facilities for PND and poor outcome for these two diseases as wells as the religious legal permission of abortion before the 16^th ^week of gestation in these genetic diseases but in relation to hemophilia nearly 60% of participants opposed to abortion may be due to better prognosis, poor feasibility for PND and unpermissionable abortion for it.

## Limitations

Limitations of our study was government disagreement with abortion for all three diseases, thus may be interfere to make decision of physician regarding abortion in these cases. But the strength point of study was well familiarity of these diseases for our physician because they are not rare in Iran.

## Competing interests

The authors declare that they have no competing interests.

## Authors' contributions

M.K drafted the manuscript and designed the study. M.M.B performed statistical analysis. M.R.G drafted the manuscript. S.Z participated in its design and coordination. All authors read and approved the final manuscript.

## Pre-publication history

The pre-publication history for this paper can be accessed here:



## Supplementary Material

Additional file 1 **Questionnaire.** It was administered to selected physicians by stratified random sampling to determine some variables. Click here for file
