# Supplementary material for: Termination of pregnancy due to Thalassemia major, Hemophilia, and Down's Syndrome: the views of Iranian physicians
Source: BMC Med Ethics. 2008 Dec 23;9:19. doi: 10.1186/1472-6939-9-19 (PMC2628659; doi:10.1186/1472-6939-9-19)
Supplement: Additional file 1 — Questionnaire. It was administered to selected physicians by stratified random sampling to determine some variables. [file 1472-6939-9-19-S1.doc]

***Questionnaire***

**Evaluation of acceptability of abortion in thalassemia major, hemophilia, and Down’s syndrome among physicians**

**- Name:**

**-Age:**

**-Gender:**

**-Status:** Intern□ Resident□ Attending□

**- Are you familiar with prenatal diagnosis of thalassemia major? Yes**□

**No**□

**Hemophilia Yes**□ **No**□

**Down’s Syndrome Yes**□ **No**□

**-Do you agree with performing an abortion for thalassemia major? Yes**□

**No**□

**Hemophilia Yes**□ **No**□

**Down’s Syndrome Yes**□ **No**□

1. **If the answer is positive :**

**Why do you agree with abortion in all of three conditions separately?**

**- Poor outcome and prognosis □**

**- Religious permission of abortion** □

**- Poor feasibility of treatment** □

**- Severity of the disease □**

**- Miscellaneous** □

**2. At what gestational age do you recommend abortion for thalassemia**

**major, hemophilia, and Down’s syndrome?**

**- <12 weeks of gestation** □

**- <16 weeks of gestation** □

**- <20 weeks of gestation □**

**- <24 weeks of gestation** □

**- >24 weeks of gestation □**

1. **If the answer is negative :**

**What are the reasons against abortion in these diseases?**

**- Religious** □

**- Emotional** □

**- Quality care for the patient** □

**- Hope to find an effective treatment in the future** □

**- Miscellaneous** □
